# ScLineageAtlas: a comprehensive single-cell genomics database for characterizing cellular clones in cancer

**DOI:** 10.1093/database/baaf046

**Published:** 2025-09-24

**Authors:** Jinyang Liu, Rui Hou, Junlin Xu, Tingting Hui, Haotian Tian, Yankun Liu, Meijun Zhang, Geng Tian, Jialiang Yang

**Affiliations:** Geneis Beijing Co., Ltd, No. 31, Laiguangying Xinbei Road, Chaoyang District, Beijing 100102, China; Qingdao Geneis Institute of Big Data Mining and Precision Medicine, No. 2, Dongshan Road, Shinan District, Qingdao 266000, China; Geneis Beijing Co., Ltd, No. 31, Laiguangying Xinbei Road, Chaoyang District, Beijing 100102, China; Qingdao Geneis Institute of Big Data Mining and Precision Medicine, No. 2, Dongshan Road, Shinan District, Qingdao 266000, China; College of Computer Science and Electronic Engineering, Hunan University, No. 2, Lushan South Road, Yuelu District, Changsha 410082, China; Geneis Beijing Co., Ltd, No. 31, Laiguangying Xinbei Road, Chaoyang District, Beijing 100102, China; Qingdao Geneis Institute of Big Data Mining and Precision Medicine, No. 2, Dongshan Road, Shinan District, Qingdao 266000, China; Beijing Zhilan Technology Co., Ltd, No. 1, Beiqing Road, Changping District, Beijing 102206, China; Department of Medical Molecular Diagnosis, Tangshan People’s Hospital, No. 65, Shengli Road, Lunan District, Tangshan, Hebei 063001, China; Tangshan Key Laboratory of Precision Medicine Testing, Tangshan People’s Hospital, No. 65, Shengli Road, Lunan District, Tangshan, Hebei 063001, China; Geneis Beijing Co., Ltd, No. 31, Laiguangying Xinbei Road, Chaoyang District, Beijing 100102, China; Qingdao Geneis Institute of Big Data Mining and Precision Medicine, No. 2, Dongshan Road, Shinan District, Qingdao 266000, China; Geneis Beijing Co., Ltd, No. 31, Laiguangying Xinbei Road, Chaoyang District, Beijing 100102, China; Qingdao Geneis Institute of Big Data Mining and Precision Medicine, No. 2, Dongshan Road, Shinan District, Qingdao 266000, China; Geneis Beijing Co., Ltd, No. 31, Laiguangying Xinbei Road, Chaoyang District, Beijing 100102, China; Qingdao Geneis Institute of Big Data Mining and Precision Medicine, No. 2, Dongshan Road, Shinan District, Qingdao 266000, China

## Abstract

Accurate identification of clonal relationships between cell populations is crucial for investigating cellular differentiation trajectories and gaining insights into the underlying mechanisms of cancer initiation and development. The Single Cell Lineage Atlas (ScLineageAtlas; https://www.scladb.geneis.org.cn) is a comprehensive single-cell genomics database that characterizes cellular clones across various cancer types. The database currently includes 24 processed single-cell RNA sequencing datasets spanning 13 different cancer types. ScLineageAtlas leverages advanced computational methods to identify cellular clones, providing researchers with a detailed understanding of clone relationships and evolutionary dynamics. Additionally, the database offers comprehensive metadata for each sample, enabling researchers to explore contextual information and sample characteristics. The spatial visualization of cell clones presented in the ScLineageAtlas provides a valuable tool for enhancing our understanding of the genetic heterogeneity within the tumour microenvironment. Through the analysis of biological differences between these diverse cell populations, researchers can explore key genes and signalling pathways associated with cancer initiation, development, and therapeutic efficacy. In summary, the ScLineageAtlas serves as a user-friendly platform for data operations on cellular clones, facilitating the understanding of tumour heterogeneity, differentiation trajectories, and evolution. It thus contributes significantly to cancer research and clinical practice.

## Introduction

The identification of cellular differentiation trajectories offers valuable insights into the mechanisms underlying cancer initiation and development [1–3]. In model organisms, lineage tracing can be accomplished by introducing heritable genetic sequences into individual cells and monitoring their alterations in descendant cells, enabling researchers to delineate the differentiation pathways of cells within tissues [4, 5]. However, the application of these methods in human studies remains impractical.

The hierarchical acquisition of genetic mutations, including single nucleotide variants (SNVs) and copy number variations (CNVs), is a fundamental characteristic of cellular clonal evolution [3, 6–8]. Despite technological advancements, the use of whole-genome sequencing to detect genetic mutations in individual cells still encounters challenges, such as high costs and elevated error rates [9]. Additionally, genetic mutations within the cell nucleus are relatively infrequent [10]. As a result, reconstructing cell lineages based exclusively on nuclear genetic information remains a significant challenge.

Tumour heterogeneity is a key factor driving tumour evolution and treatment resistance [11, 12]. Different subclones respond variably to targeted therapies, and resistant subclones can expand under treatment pressure, leading to disease progression. Furthermore, even tumours of the same pathological type can exhibit different molecular characteristics in different patients. Therefore, personalizing treatment based on the unique tumour features of each patient can significantly improve treatment success rates.

Mutations in mitochondrial DNA (mtDNA) are effective endogenous genetic markers for reconstructing cellular clonal structures [13–15]. mtDNA mutations are prevalent across various tumours and are considered key factors influencing tumour heterogeneity. Research indicates that the burden of mtDNA mutations exhibits significant lineage specificity among different tumour types, with certain cancers showing markedly higher mutation rates than others. These mutations not only impact cellular metabolic homeostasis but may also exacerbate tumour heterogeneity by promoting the clonal evolution of tumour cells [16]. Kwok et al. [14] introduced MQuad, a method for identifying mitochondrial variants (mtSNVs) from single-cell RNA sequencing (scRNA-seq) and the assay for transposase-accessible chromatin with high throughput sequencing (ATAC-Seq) data for clonality inference. However, the integration of large-scale scRNA-seq data presents significant challenges, including the need for substantial data processing expertise and computational resources, as well as addressing the presence of batch effects. Therefore, establishing a platform capable of comprehensively characterizing cellular clones by integrating published data across studies is critically needed.

Recent studies have also shown that clonal evolution exhibits significant heterogeneity [16]. Clonal evolution is considered one of the most critical aspects of cancer and plays a key role in its treatment. The ability to identify new clones, particularly at their emergence, and to determine which therapeutic strategies are effective against these clones will become increasingly important in clinical settings [17]. This understanding can potentially guide more personalized and effective treatment approaches, ultimately improving patient outcomes.

Here, we present Single Cell Lineage Atlas (ScLineageAtlas), a manually curated single-cell clone database for human tumours. By integrating publicly available scRNA-seq data and characterizing clones across various cancers, ScLineageAtlas elucidates clonal relationships and evolutionary patterns, enhancing our understanding of tumour heterogeneity and differentiation.

## Materials and methods

### scRNA-seq data collection

We systematically collected scRNA-seq data by searching PubMed for the literature related to scRNA-seq research using ((cancer[Title/Abstract]) OR (tumour[Title/Abstract])) AND ((single cell RNA sequencing[Title/Abstract]) OR (scRNA-seq [Title/Abstract])) as keywords. The literature was then manually confirmed whether the raw FASTQ data generated by the CellRanger mkfastq command were publicly available from the sequence read archive (SRA) repository. We downloaded the data from SRA and manually mapped FASTQ files to the corresponding samples. Through a manual review of all relevant literature and supplemental materials, we collected clinical information for each dataset, including patient age, gender, and tissue type. Additionally, we provided details on the sequencing platforms and library preparation methods, encompassing platform type, library strategy, source, and selection criteria. For comprehensive metadata regarding the samples, refer to Supplementary Table 1.

### Genotyping single cells

The pipeline genotypes each cell in scRNA-seq data through many steps. The input data consist of the FASTQ files collected by the above procedure. Then, BAM files are generated using the CellRanger count command. The cellSNP-lite [18], a C/C++-based tool, efficiently genotypes bi-allelic single-nucleotide polymorphisms (SNPs) in single cells. We utilized cellSNP-lite (v1.2.1) to conduct pileup analysis on raw reads from BAM files, generating two output types: (1) an SNP-by-cell matrix in VCF format and (2) a sparse matrix detailing allele depth (AD) and total sequencing depth (DP) for each cell at variant sites. Importantly, the output includes all SNPs detected in the mitochondrial genome, which is characterized by significant technical noise and non-informative variants. Throughout the analysis, cellSNP-lite was operated with default parameters.

### Quality control on inferred variant data and clonal assignments

The SNP-by-cell matrix includes all SNPs located in the mitochondrial genome derived from raw reads, resulting in significant noise and many uninformative variants. To address this, we employed MQuad (v0.1.6) to identify high-quality, informative variants using a binomial mixture model. Compared to Gaussian mixture models, the binomial mixture model incorporates heterogeneity as a proportional parameter and directly utilizes raw read counts. In this model, the number of reads (or UMIs) supporting the alternative allele (AD) at a given SNP is assumed to follow a binomial distribution, where the total number of trials equals the combined depth of both alleles (DP), and the success probability depends on variant presence. MQuad utilizes a model selection approach to evaluate the informativeness of each variant, providing a more robust analysis than relying solely on raw allele frequencies. It assesses the heteroplasmy of mtDNA variants through this binomial mixture model, which treats heteroplasmy as a proportional value and can directly utilize raw read counts. MQuad identifies the optimal ‘knee’ point in the distribution of SNPs to detect outlier SNPs with the highest clonal discriminative power, enabling the robust identification of genetically distinct cellular populations. Finally, vireoSNP17 (v0.5.3) conducts variational inference to cluster cells into clones based on the SNPs selected by MQuad.

### Quality control and batch effect correction on scRNA-seq data

The expression matrix of scRNA-seq could be downloaded from the Gene Expression Omnibus, the website provided by the original paper, or the output of the CellRanger count command. Using the quality control parameters and scanpy (v1.8.2), we filtered low-quality cells and rarely expressed genes. For each dataset, after normalization to UPM (UMI per million) and integrating across samples, the batch effect removal was performed using Harmony and BBKNN [19].

### Cell clustering and annotation

BBKNN constructs a k-nearest-neighbour graph for the whole dataset considering the batch effect. Using this graph, scanpy can perform Leiden, an unsupervised graph-based clustering method, to cluster cells. The cell-type annotation of scRNA-seq data is processed following the methods described in the corresponding paper. Sometimes, the metadata provided by the original authors already contain the cell-type annotations and we prefer to use them instead of re-doing the whole procedure.

### Visualization of individual cells

Cell-type visualization of a dataset was performed using both t-distributed stochastic neighbour embedding and the uniform manifold approximation and projection (UMAP) method. UMAP was generated by the graph from BBKNN.

### NEBULA analysis of scRNA-seq data

The negative binomial mixed model using large sample approximation (NEBULA) is a new fast algorithm for differential gene expression analysis of scRNA-seq data [20]. Using the single-cell data and the clone labels as input, we discern the differentially expressed genes (DEGs) between two specific clones within a particular cell type.

### Database construction

The front-end of the ScLineageAtlas website was developed using Vue 3.3.0 and Element Plus version 2.3.5. The back-end of the website was developed using Java and Spring Boot. Data storage and management were performed using MySQL version 5.7. The ECharts version 5.4.2 plugin software was utilized to create interactive tables and visualize the results. All upstream and downstream analyses were performed using R version 4.1.2 and Python version 2.7, based on the Linux operating system.

## Results

### Overview of ScLineageAtlas

**Figure 1.**
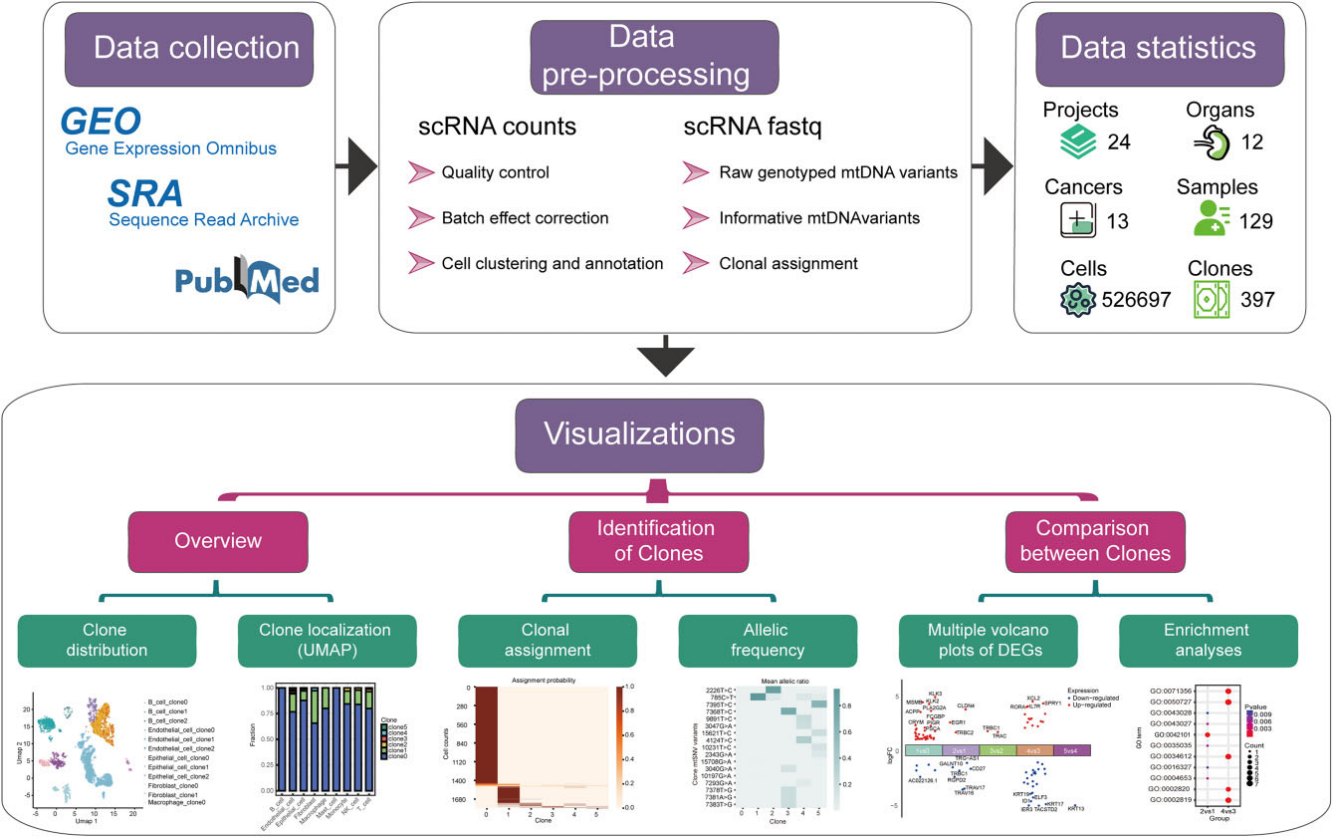
Scheme of the ScLineageAtlas portal.

ScLineageAtlas employing a standardized workflow that includes quality control, normalization, batch correction, and meticulous manual adjustment of meta-information, coupled with an integrated clonality discovery pipeline cellSNP-MQuad-VireoSNP effectively identifies mtDNA variants and infers clonal relationships between cells (Fig. 1). This comprehensive platform provides a suite of visualization tools that facilitate the elucidation of clonal structures and evolutionary patterns within cancer tissues.

### Summary of datasets in ScLineageAtlas

**Figure 2.**
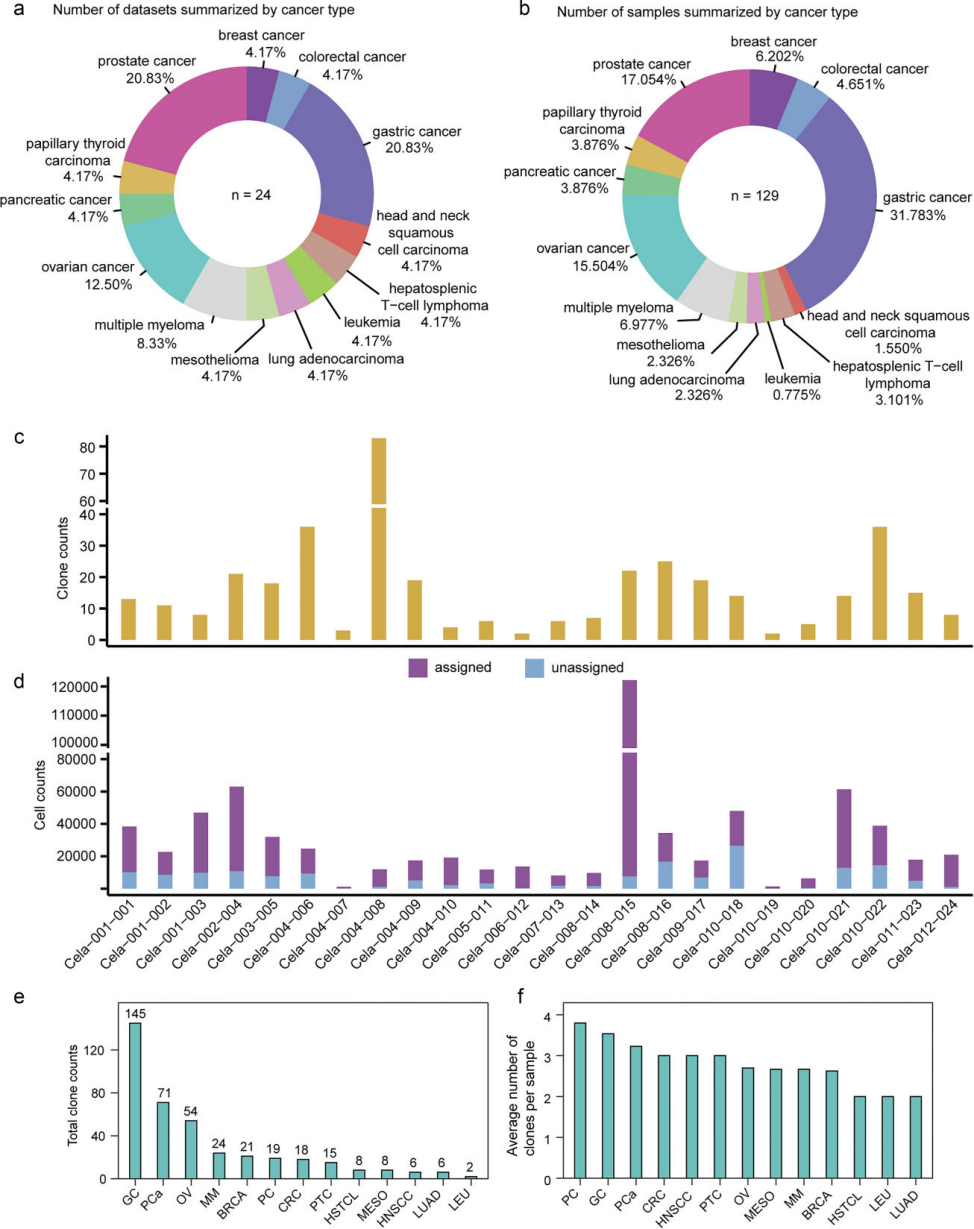
Statistics in ScLineageAtlas. (a) Number of datasets summarized by cancer type. (b) Number of samples summarized by cancer type. (c) Clone counts summarized by dataset. (d) Assigned and unassigned cell counts summarized by dataset. (e) and (f) illustrate the distribution of total clone numbers and average clone numbers per sample across different cancer types, respectively. PC: pancreatic cancer; OV: ovarian cancer; CRC: colorectal cancer; GC: gastric cancer; HNSCC: head and neck squamous cell carcinoma; PTC: papillary thyroid carcinoma; PCa: prostate cancer; BRCA: breast cancer; HSTCL: hepatosplenic T-cell lymphoma; LEU: leukaemia; LUAD: lung adenocarcinoma; MESO: mesothelioma; and MM: multiple myeloma.

Currently, the database comprises 24 datasets and 129 samples across 13 cancer types (Fig. 2a and b). The total number of clones is 397, ranging from two to six across various datasets (Fig. 2c). The number of cells assigned to clones totals 526 697, ranging from 90 to 18 512 across various datasets (Fig. 2d). In ScLineageAtlas, the three cancers with the highest total number of clones are gastric cancer, prostate cancer, and ovarian cancer (Fig. 2e). Conversely, the cancers with the highest average number of clones per sample are pancreatic cancer, followed by gastric cancer and prostate cancer (Fig. 2f). Pancreatic cancer is a highly lethal malignancy characterized by significant inter- and intra-tumoural heterogeneity [21, 22].

### Comparison with other mainstream single-cell genomics databases

We have systematically compiled and analysed ScLineageAtlas alongside existing databases, examining various dimensions such as primary functionality, biological focus, and database scale. The results of this comparison are presented in Table 1.

**Table 1.** Comparison of ScLineageAtlas with other mainstream single-cell genomics databases.

Database	Main functionality	Biological focus	Cell count	Dataset count	Disease/cancer types	Species
scMethBank	Single-base resolution single-cell DNA methylation profiling	Epigenetic heterogeneity, cell state regulation	8328	15	2	Human, mouse
scCancerExplorer	Integrated analysis and visualization of single-cell multi-omics data (genome + epigenome + transcriptome)	Pan-cancer molecular mechanisms, multi-omics correlations	6200 000	161	50	Human
HSCGD	Single-cell whole-genome mutation profiling (SNVs/CNVs)	Genomic instability, somatic mutation accumulation	74 154	63	8	Human
ScLineageAtlas	Clonal lineage reconstruction and evolutionary analysis at single-cell resolution	Tumour clonal evolution, heterogeneity, and differentiation	526 697	24	13	Human

ScLineageAtlas is the first platform to analyse tumour heterogeneity through the lens of clonal dynamics. Its lineage tracing feature offers an essential tool for investigating the mechanisms of treatment resistance and metastasis.

### User-friendly searching modules to efficiently retrieve data

**Figure 3.**
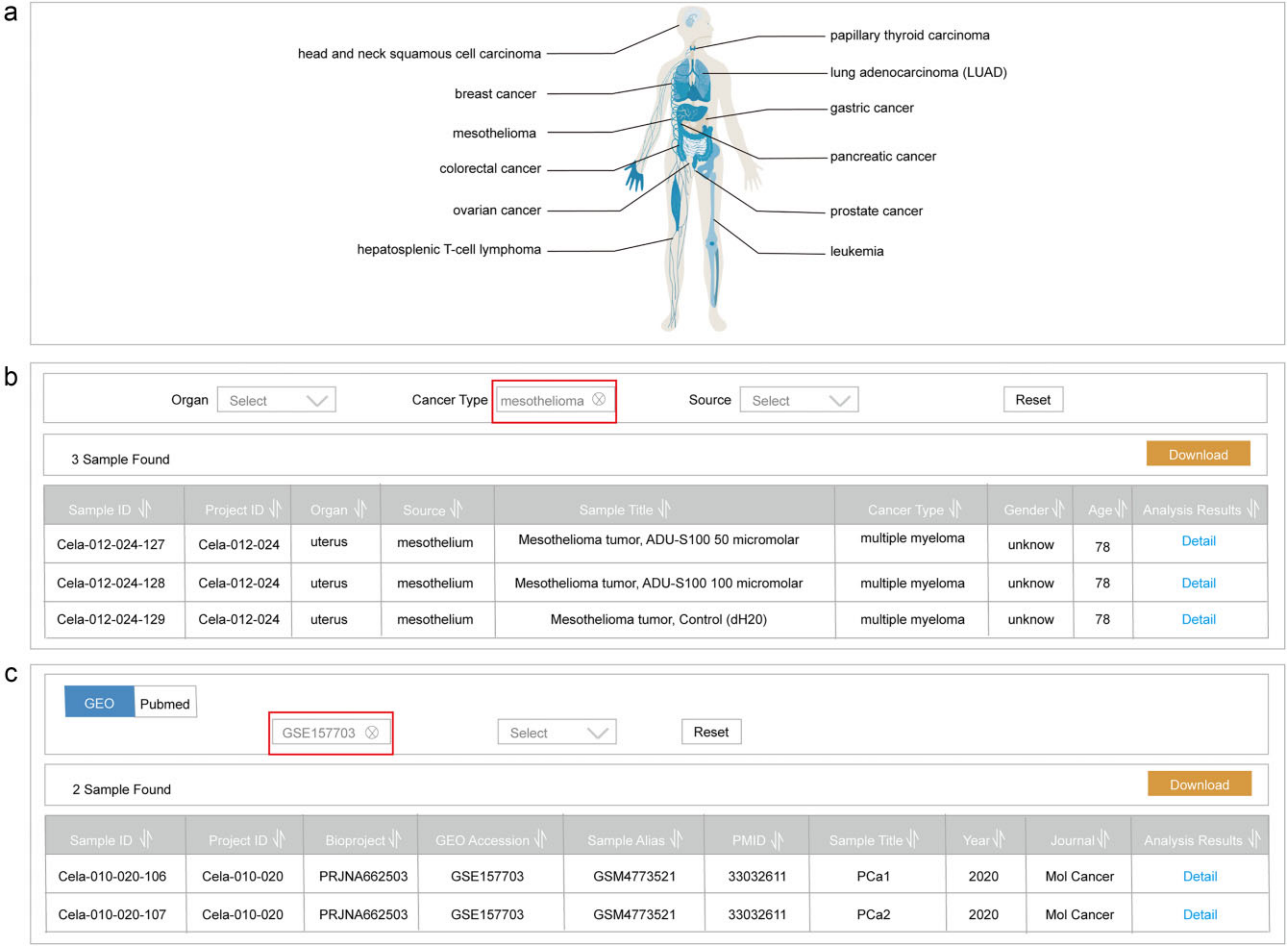
Data retrieval functionality. (a) Interactive human organism maps visually representing cancer type information; (b) a search interface based on clinical information; and (c) a search interface based on reference metadata.

The home page of ScLineageAtlas utilizes interactive human organism maps to visually represent the cancer type information stored in the database (Fig. 3a). Users are able to click on the organ icon for direct navigation to the corresponding dataset of interest. Besides visual navigation, the home page also provides a search function, enabling users to query the database with multiple parameters, including organ, cancer type, bioproject ID, the Gene Expression Omnibus (GEO) accession, and sample alias.

The ScLineageAtlas platform also provides a dedicated ‘Search’ page for users to quickly obtain the information of interest. This search interface includes two distinct approaches: searching based on clinical information (Fig. 3b) and searching based on reference metadata (Fig. 3c). To facilitate more targeted searches, users can utilize a drop-down menu to select specific criteria, such as organ, cancer type, or data source. All search results are efficiently displayed in a tabular format and are readily downloadable.

### Comprehensive multiple-dimensional online data exploration

ScLineageAtlas provides users with eight interactive modules designed to facilitate a comprehensive and in-depth exploration of clone relationships and evolutionary dynamics. By clicking the ‘Detail’ button within the search result table, users can access all the analysis results for their selected samples.

#### Exploring the spatial distribution of clones

**Figure 4.**
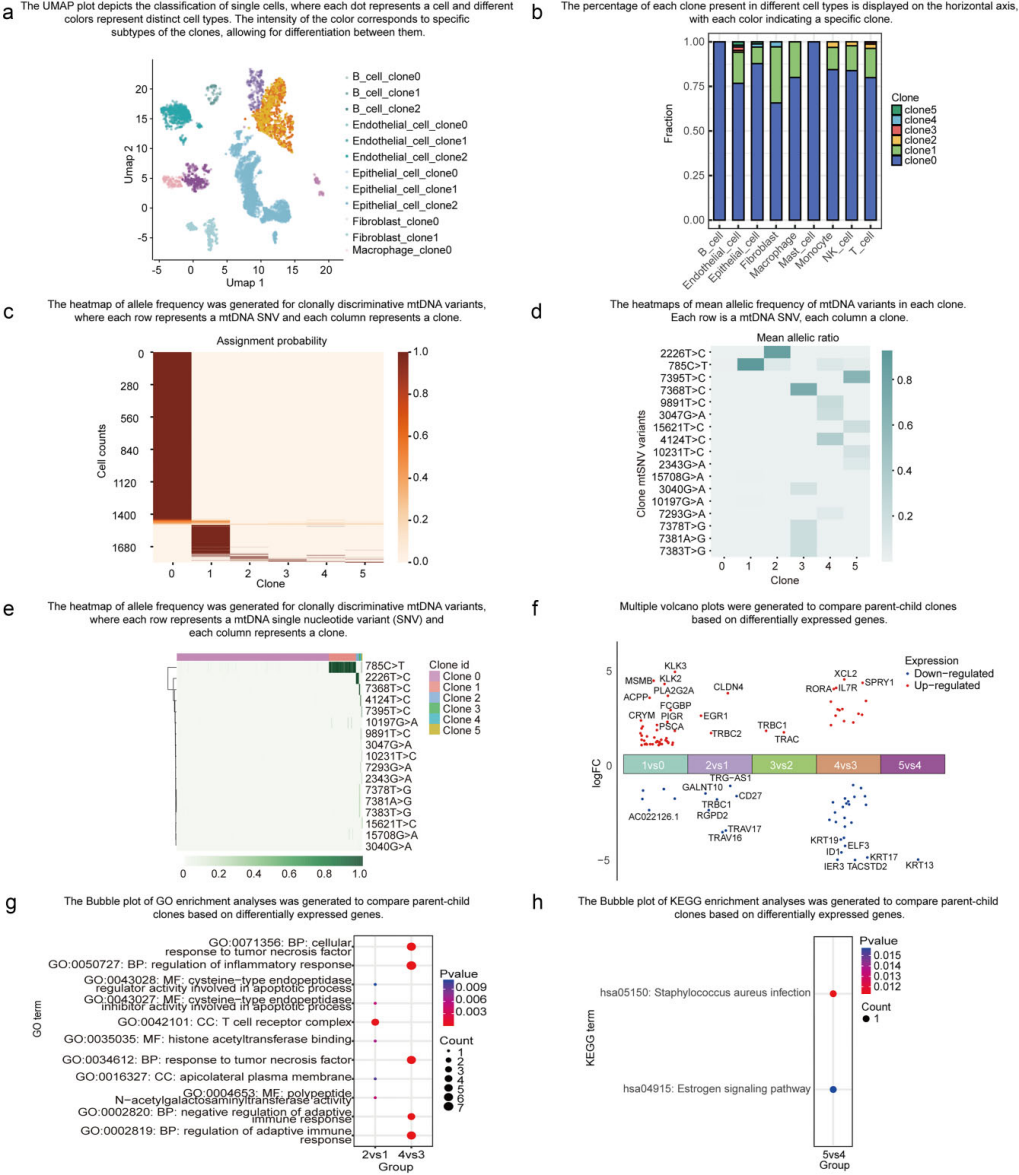
Data exploration functionality. (a) UMAP of cell types, where each dot represents a cell and different colours indicate distinct cell types. (b) Bar plot displaying clone fractions in different cell types. (c) Heatmap showing the probability of each cell being assigned to each clone, where each row represents a cell and each column represents a clone. (d) Heatmap of mean allelic frequency of mtDNA variants in each clone, with each row representing an mtDNA SNV and each column representing a clone. (e) Heatmap of allele frequency for clonally discriminative mtDNA variants, where each row represents an mtDNA SNV and each column represents a cell. (f) A volcano plot comparing two specific clones was generated based on DEGs. (g) A bubble plot illustrating the Gene OO)enrichment analysis for the two clones based on DEGs. (h) A bubble plot displaying the Kyoto Encyclopedia of Genes and Genomes (KEGG) enrichment analysis for the two clones based on DEGs.

ScLineageAtlas empowers users to explore the spatial distribution of clones through the readily accessible ‘Overview’ menu. The platform delivers two key visualizations to facilitate this crucial analysis. The UMAP module depicts the classification of individual cells in a visually compelling manner (Fig. 4a). Distinct colours are utilized to represent the different cell types, with the intensity of the colour corresponding to a specific clone subtype. The Clone Abundance Module displays the percentage of each clone within the specific cell type (Fig. 4b). Each colour within this module signifies a distinct clone, providing invaluable insights into the relative abundance of each clone across the multifarious cell types. These interactive visualizations empower users to gain a comprehensive understanding of the spatial distribution and clonal composition of the samples within the ScLineageAtlas.

#### Evaluating genetic heterogeneity

The ‘Identification of Clones’ menu provides users with access to critical information pertaining to cell clone assignment probabilities and allele frequencies of clonally informative mtDNA variants. The Cell Assignment Probability module presents a visualization of the likelihood of each cell being assigned to a specific clone (Fig. 4c), empowering researchers to assess the confidence of the cell assignments. The Mean mtSNV Allelic Frequency module (Fig. 4d) and mtSNV Allelic Frequency module (Fig. 4e) decipher the mtSNV mutational profiles of individual clones, allowing users to identify highly clone-specific variants and evaluate the extent of genetic heterogeneity. These modules offer invaluable insights into the characteristics and genetic makeup of the individual clones within the analysed samples.

#### Characterization of biological significance across different cell populations

Functional characterization at the gene and pathway levels of individual clones can shed invaluable light on the mechanisms driving tumour progression and relapse. By utilizing the ‘Comparison between Clones’ menu, users can access the results of DEGs and pathways enrichment analysis across different clones (Fig. 4f–h). These advanced features empower users to conduct rigorous comparative assessments of the biological significance among distinct cell populations.

### Example application

To demonstrate the utility and potential applications of ScLineageAtlas, we analysed Cela-010-022-119 (tumour sample) and Cela-010-022-121 (benign sample) from the same patient with prostate cancer. In the tumour sample, we identified a highly clone-specific mutation, 785C>T, in clone 1, which was also observed in normal samples, suggesting that clone 1 may represent a progenitor clone. Additionally, novel mutations were detected in clone 2 (2226T>C), clone 3 (7368T>C), clone 4 (4124T>C), and clone 5 (7395T>C) within the tumour sample, indicating that these cell populations may represent novel clones. These novel clones are predominantly found in endothelial cells, epithelial cells, fibroblasts, T cells, monocytes, and NK cells. Furthermore, through the examination of DEGs between the mtDNA clones, we identified DEGs in specific subclones. For instance, when compared with clone 1, the expression of EGR1 is up-regulated in clone 2. EGR1 plays a significant role in promoting the progression and metastasis of prostate cancer [23, 24]. Ho et al. [25] elucidated the critical regulatory role of EGR1 in renal inflammation and fibrosis, suggesting its potential as a therapeutic target for human renal diseases. Additionally, research has demonstrated that EGR1 can inhibit cholestasis-induced hepatic inflammatory responses [26], highlighting its significant value in the treatment of liver injury. In comparison with clone 3, the expression of ELF3 is down-regulated in clone 4. ELF3 activates NF-kB signalling pathway and drives prostate cancer [27]. Studies have demonstrated that ELF3 expression levels are significantly correlated with tumour metastatic potential [28]. Numerous studies have indicated that ELF3 plays a regulatory role in mesenchymal–epithelial transition and epithelial–mesenchymal transition (EMT) [29, 30]. EMT enhances the migratory and invasive capabilities of tumour cells, facilitating their dissemination from primary tumours to distant sites. Additionally, EMT is often associated with the acquisition of cancer stem cell properties, which contributes to increased therapeutic resistance. Gene Ontology (GO) enrichment analysis demonstrates that the pathways related to adaptive immunity, tumour necrosis factor, and inflammatory response are dysregulated during clonal evolution.

## Summary and future developments

As a freely and openly accessible database platform, ScLineageAtlas enables researchers to explore clonal evolution in human tumours. It integrates multi-source scRNA-seq and offers cell-type annotations along with visualizations of differential gene analysis results, all based on a standardized analytical workflow that includes quality control, dimensionality reduction, and clustering. These features address the needs of fundamental research while providing open data downloads and an interactive analysis interface, thereby significantly reducing the operational barriers for non-bioinformatics users. In contrast to other databases, ScLineageAtlas specifically focuses on the genetic heterogeneity of cancer cell clones, enhancing our understanding of tumour heterogeneity, differentiation trajectories, and evolution. Consequently, it makes substantial contributions to both cancer research and clinical practice.

Its update plan encompasses the following aspects: Future versions will centre on multi-omics integration, including genomics, transcriptomics, and epigenomics. Currently, ScLineageAtlas is limited to mitochondrial variants due to the technical challenges in detecting low-frequency mutations from scRNA-seq data. With the advancements in single-cell genomics techniques, nuclear mutations will be incorporated into our analyses, and several computational tools have already been developed for lineage inference, utilizing CNVs, nuclear DNA mutations, and mtSNVs. Notable examples include Cardelino [6], LineageOT [31], PhylEx [3], MutaSeq [32], and EMBLEM [13]. We plan to systematically evaluate and compare the performance of these tools to inform the development of next-generation lineage inference methods based on our findings. Furthermore, beyond cancer research, elucidating lineage relationships between cells is crucial for understanding the cellular origins of disease development, determining cell fate during embryonic development, and mapping the dynamic trajectories of tissue and organ formation. This knowledge offers essential insights into the complexity of life processes and the fundamental mechanisms underlying disease emergence. Therefore, we plan to expand our dataset to encompass a wider range of disease conditions and tissue types. Additionally, we intend to introduce a ‘Compare’ page, which will allow users to conduct cross-dataset comparisons, facilitating the analysis of gene expression alterations and pathway activities among samples across various cancer types and tissues. We will also provide batch-corrected data using multiple correction techniques, enabling users to select the method that best fits their research needs. These enhancements aim to improve the functionality and usability of ScLineageAtlas, ultimately facilitating a deeper understanding of clonal relationships and evolutionary patterns.

## Supplementary Material

baaf046_Supplemental_File

## Data Availability

All public datasets we gathered in ScLineageAtlas are available from GEO. All public datasets that were processed and integrated into the database are available at https://www.scladb.geneis.org.cn, with no login requirement.
